# Using a smartphone-based self-management platform to study sex differences in Parkinson’s disease: multicenter, cross-sectional pilot study

**DOI:** 10.1186/s12911-024-02569-1

**Published:** 2024-06-21

**Authors:** Zhiheng Xu, Lirong Jin, Weijie Chen, Tianyu Hu, Shiyu Li, Xiaoniu Liang, Xixi Han, Yi Chen, Yilin Tang, Jian Wang, Danhong Wu

**Affiliations:** 1 Department of Neurology, Shanghai Fifth People’s Hospital, Fudan University, 801 Heqing Road, Shanghai, 200240 China; 2 Department of Neurology and National Research Center for Aging and Medicine & National Center for Neurological Disorders, State Key Laboratory of Medical Neurobiology, Huashan Hospital, Fudan University, Shanghai, 200040 China; 3 Department of Neurology, Zhongshan Hospital, Fudan University, Shanghai, 200032 China

**Keywords:** Parkinson’s disease, mHealth, Patient-reported outcomes, Sex differences

## Abstract

**Background:**

Patient-reported outcome (PRO) is a distinct and indispensable dimension of clinical characteristics and recent advances have made remote PRO measurement possible. Sex difference in PRO of Parkinson’s disease (PD) is hardly extensively researched.

**Methods:**

A smartphone-based self-management platform, offering remote PRO measurement for PD patients, has been developed. A total of 1828 PD patients, including 1001 male patients and 827 female patients, were enrolled and completed their PRO submission through this platform.

**Results:**

Sex differences in PROs have been identified. The female group had a significantly lower height, weight, and body mass index (BMI) than the male group (*P* < 0.001). For motor symptoms, a higher proportion of patients reporting dyskinesia was observed in the female group. For non-motor symptoms, there is a higher percentage (*P* < 0.001) as well as severity (*P* = 0.016) of depression in the female group. More male patients reported hyposmia, lisp, drooling, dysuria, frequent urination, hypersexuality, impotence, daytime sleepiness, and apathy than females (*P* < 0.05). In contrast, more female patients reported headache, palpation, body pain, anorexia, nausea, urinal incontinence, anxiety, insomnia (*P* < 0.05) than males.

**Conclusions:**

We provide evidence for sex differences in PD through the data collected from our platform. These results highlighted the importance of gender in clinical decision-making, and also support the feasibility of remote PRO measurement through a smartphone-based self-management platform in patients with PD.

**Supplementary Information:**

The online version contains supplementary material available at 10.1186/s12911-024-02569-1.

## Introduction

Patient-reported outcome (PRO) is defined by the US Food and Drug Administration as ‘a measurement of any aspect of a patient’s health status that comes directly from the patient, without the interpretation of the patient’s responses by a physician or anyone else’ [1]. Thus, it has been widely applied for severity evaluation and included in clinical trial protocols. As it captures a patient’s perception of certain aspects, PRO has revealed a distinct yet indispensable dimension of certain disease’s clinical characteristics [2].

In Parkinson’s disease (PD), it has long been suggested that there exist sex differences, which is strongly supported by the fact that the prevalence and incidence of PD are lower in women than men [3]. These distinctions can present beyond epidemiology, and support the idea that disease development might involve distinct pathogenic mechanisms in male and female patients [3–6]. Increased recognition of these differences shall bring a more individualized clinical management for patients and a better design of clinical studies and trials. Therefore, it would be helpful to explore sex differences in PD.

Although sex difference also exists in PROs, it is hardly extensively reviewed and recognized in previous researches. In recent years, rapid advances in portable devices and communication technologies have spurred the emergence of mobile health (mHealth), which in turn made remote PRO measurement possible. The utility of remote assessment tools has revolutionized our way of data collection and has shown great promise in increasing the feasibility of conducting comprehensive evaluation and longitudinal tracking.

We have previously developed a smartphone-based self-management platform which offers a set of established questionnaires and binary questions aiming to collect PROs remotely [7]. Based on the data collected, we here present our preliminary multicenter, cross-sectional pilot study on sex differences in PD and demonstrate the feasibility of remote PRO measurement in patients with PD.

## Methods

### Study population

PD was diagnosed by a movement disorders specialist according to the 2015 MDS Clinical Diagnostic Criteria [8] for PD during an in-clinic evaluation. PD patients who owned IOS or Android smartphones were invited to download ‘Pawei’ app. developed by our group as a not-for-profit effort to support the management of PD remotely (Pawei – which is Chinese for ‘for Parkinson’s disease’). At the time of the start of this present study (January 2017), 126 hospitals across China had joined this platform. Details about the app have been reported [7]. Participants were provided with instructions on how to use the app. The methods were performed in accordance with relevant guidelines and regulations and approved by the Human Studies Institutional Review Board, Huashan Hospital, Fudan University.

### Data acquisition

Following consent, participants are asked to complete a set of established questionnaires and binary questions at baseline and each season (at 90-day intervals) for regular evaluation as their PROs. These questions are designed to depict the patient’s perception of certain items, which can mainly be categorized into three dimensions: motor symptoms, non-motor symptoms, and life quality. Questionnaires include the Movement Disorder Society-Sponsored Revision of the Unified Parkinson’s Disease Rating Scale (MDS-UPDRS IB and II) [9], Non-motor Symptoms Scale (NMSS) [10], Beck Depression Inventory (BDI) [11] and REM Sleep Behavior Disorder Screening Questionnaire (RBDSQ) [12] and Parkinson Disease Questionnaire 8 (PDQ-8) [13, 14]. Questions are designed as a dichotomous scale, where a patient can indicate whether they are afflicted or not by the particular symptom by submitting ‘Yes’ or ‘No’ as their answer. In addition, patients were instructed to complete information about current medication use (duration and doses of PD drugs, possible use of any drugs known to cause drug-induced parkinsonism, and any other drugs). The dosage of anti-parkinsonian drugs was converted into a total daily levodopa equivalent dose (LED) [15].

We enrolled all patients who had already been diagnosed as PD in the movement specialist clinic and also completed PRO submission at least one time between January 2017 and March 2021 through this app. For patients who have completed the self-evaluation several times, we chose the baseline data to represent the patient’s PROs.

### Data security

Regarding data security in this platform, all data are transferred using hypertext transfer protocol, HTTP, within a connection encrypted by transport layer security or secure sockets layer. The HTTPS encryption is performed prior to any HTTP communication, so the whole interaction is protected. The endpoints’ security is also risk-managed by storing audit logs generated by every module of the platform, allowing usage tracking of modules and users. The client/user authentication interfaces are implemented following the OAuth 2.0 authorization framework specifications, an open protocol that enables secure authorization for web-based, desktop, and mobile applications. Finally, access control management is implemented using a user authorization system that is responsible for defining the content that each user is allowed to access and manage.

### Statistical analysis

Categorical variables were expressed as frequencies (%), and the Chi-squared test was used to compare the categorical variables. As for continuous variables, we used mean ± standard deviation (SD) to report the results, and Wilcoxon signed-rank test was used to compare the continuous variables due to the data was not normally distributed (Shapiro–Wilk test: *p* < 0.05). Two-tailed *p* values are presented, and differences were considered statistically significant at *p* < 0.05. R version 4.1.0 was used for the analysis.

## Results

### Basic clinical characteristics

Of 1828 enrolled PD patients, 1001 male and 827 female were included in the analysis. Basic clinical characteristics are described in Table 1. There were no differences in age, age at onset, disease duration and LED between the two groups. The female group showed a shorter number of education years than the male group (*P* < 0.05). Besides, the female group had a significantly lower height, weight, as well as BMI, compared to the male group (*P* < 0.001).

**Table 1:** Basic clinical characteristics of patients

Variables	Total (*N* = 1828)	Male (*N* = 1001)	Female (*N* = 827)	*P* Value*
Age, year, mean ± std	61.1 ± 11.6	61.0 ± 12.0	61.1 ± 11.0	0.901
Age at onset, year, mean ± std	59.2 ± 11.6	59.2 ± 12.1	59.1 ± 11.1	0.495
Disease duration, year, mean ± std	1.9 ± 3.3	1.8 ± 3.1	2.0 ± 3.6	0.327
Height, m, mean ± std	1.7 ± 0.8	1.7 ± 0.5	1.6 ± 0.6	< 0.001†
Weight, kg, mean ± std	64.0 ± 11.2	69.0 ± 9.4	57.6 ± 10.0	< 0.001†
BMI, kg/m2, mean ± std	23.2 ± 3.3	23.7 ± 2.8	22.6 ± 3.7	< 0.001†
Education, year, mean ± std	11.7 ± 4.4	12.2 ± 3.9	11.1 ± 4.9	0.020†
LED, mg/day, mean ± std	515.9 ± 482.4	518.4 ± 464.5	512.9 ± 503.4	0.364

Abbreviations: BMI: body mass index; LED: levodopa equivalent dose

Note: *Comparison between the male and female group. † Two-tailed *p* values are presented, and differences were considered statistically significant at *p* < 0.05

### PROs by sex

For questionnaires (Table 2), a comparison of the MDS-UPDRS IB score, MDS-UPDRS II score, NMSS score, RBDSQ score, and PDQ-8 score yielded no significant difference between the two groups. However, the female group presented a significantly higher BDI score (14.5 vs. 11.6, *P* = 0.016), indicating a higher severity of depression. Linear regression analysis between LED and clinical questionnaire scores by sex are listed in the Supplementary Data.

**Table 2:** Clinical questionnaire scores by sex

Variables	Total	Male	Female	*P* Value*
MDS-UPDRS IB, score, mean ± std	7.1 ± 4.9	7.0 ± 4.9	7.1 ± 4.9	0.669
MDS-UPDRS II, score, mean ± std	11.6 ± 7.4	11.9 ± 7.4	11.3 ± 7.3	0.148
NMSS, score, mean ± std	28.4 ± 32.2	26.4 ± 27.3	30.8 ± 37.5	0.896
BDI, score, mean ± std	12.9 ± 9.3	11.6 ± 9.3	14.5 ± 9.1	0.016†
RBDSQ, score, mean ± std	4.4 ± 3.4	4.4 ± 3.6	4.3 ± 3.2	0.985
PDQ-8, score, mean ± std	9.7 ± 6.9	9.7 ± 6.8	9.8 ± 6.9	0.797

Abbreviations: PDQ-8: Parkinson Disease Questionnaire 8; BDI: Beck Depression Inventory; NMSS: Non-motor Symptoms Scale; MDS-UPDRS: Movement Disorder Society-Sponsored Revision of the Unified Parkinson’s Disease Rating Scale; RBDSQ: REM Sleep Behavior Disorder Screening Questionnaire

Note: *Comparison between the male and female group. † Two-tailed *p* values are presented, and differences were considered statistically significant at *p* < 0.05

**Fig. 1:**
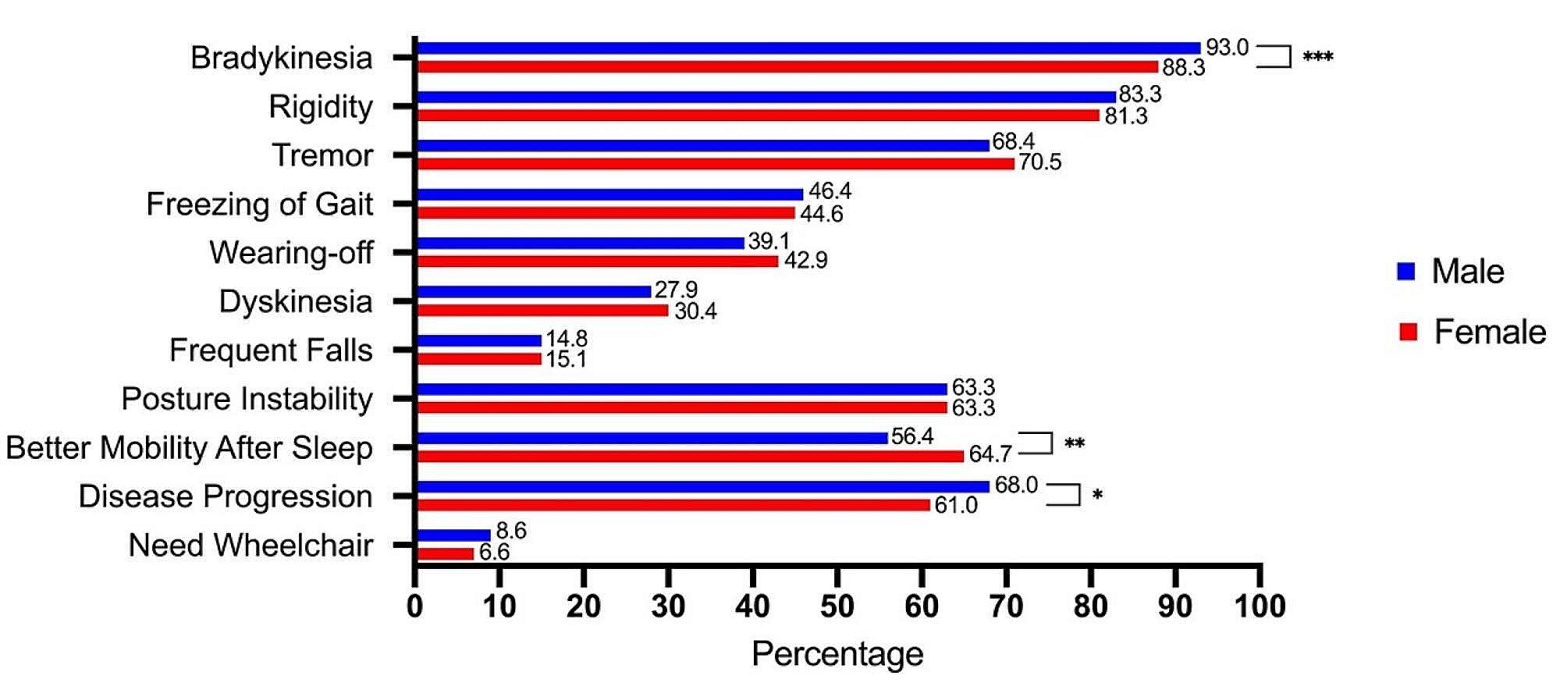
Motor symptoms reported by sex

**Fig. 2:**
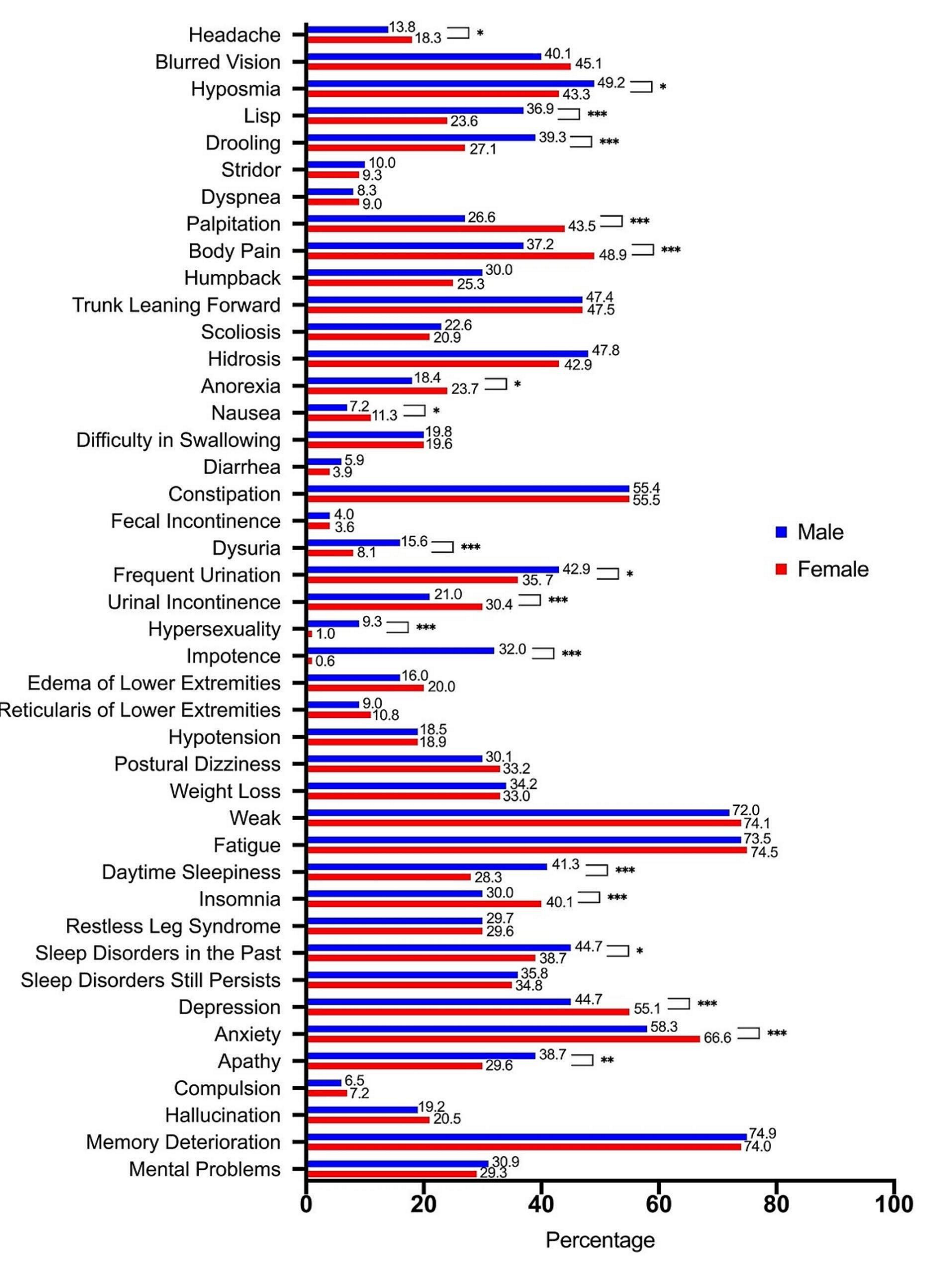
Non-motor symptoms reported by sex

In addition, through a series of binary questions, we have identified several differences in respect to sex. For motor symptoms (Fig. 1), more male group reported bradykinesia (93.0% vs. 88.3%, *P* < 0.001) and disease progression (68.0% vs. 61.0%, *P* = 0.016), but less recovery from sleep (56.4% vs. 64.7%, *P* = 0.002). More female patients reported wearing-off phenomenon (42.9% vs. 39.1%) and dyskinesia (30.4% vs. 27.9%). For non-motor symptoms (Fig. 2), male group presented a statistically higher proportion of positive answers in hyposmia (49.2% vs. 43.3%, *P* = 0.021), lisp (36.9% vs. 23.6%, *P* < 0.001), drooling (39.3% vs. 27.1%, *P* < 0.001), dysuria (15.6% vs. 8.1%, *P* < 0.001), frequent urination (42.9% vs. 35.7%, *P* = 0.013), hypersexuality (9.3% vs. 1.0%, *P* < 0.001), impotence (32.0% vs. 0.6%, *P* < 0.001), daytime sleepiness (41.3% vs. 28.3%, *P* < 0.001), claiming suffering sleep disorders in the past (44.7% vs. 38.7%, *P* = 0.014) and apathy (48.7% vs. 29.6%, *P* = 0.001). Female group instead presented a statistically higher proportion of positive answers in headache (18.3% vs. 13.8%, *P* = 0.035), palpation (43.5% vs. 26.6%, *P* < 0.001), body pain (48.9% vs. 37.2%, *P* < 0.001), anorexia (23.7% vs. 18.4%, *P* = 0.023), nausea (11.3% vs. 7.2%, *P* = 0.013), urinal incontinence (30.4% vs. 21.0%, *P* < 0.001), insomnia (40.1% vs. 30.0%, *P* < 0.001), depression (55.1% vs. 44.7%, *P* < 0.001) and anxiety (66.6% vs. 58.3%, *P* < 0.001) than males.

Patients were asked to indicate whether they are afflicted or not by the particular symptom by submitting ‘Yes’ or ‘No’ as their answer, and the percentage of positive answers by gender is shown here. A comparison was made between the male and female groups, and a *P* value was calculated: *P < 0.05; **P < 0.01; ***P < 0.001.

Patients were asked to indicate whether they are afflicted or not by the particular symptom by submitting ‘Yes’ or ‘No’ as their answer, and the percentage of positive answers by gender is shown here. A comparison was made between the male and female groups, and a *P* value was calculated: *P < 0.05; **P < 0.01; ***P < 0.001.

### PROs by depression and non-motor impairment severity

We have also conducted PROs comparison between patients with depression and patients without depression; patients with high NMSS score and patients with low NMSS score (Supplementary Data).

## Discussion

To our knowledge, this is the first study to study sex differences in PROs through a smartphone-based self-management platform, demonstrating remote PRO measurement’s feasibility in PD patients. We have analyzed sex differences in PD based on the data we have collected from a total of 1828 verified patients, which is one of the largest populations studied on PROs in PD. Our results suggested that patients’ perception of certain symptoms in PD can differ substantially by sex. The actual understanding of the mechanisms underlies such differences required further researches. Nevertheless, it is of great importance to recognize such distinction, thus enabling better clinical management, both for women and men.

The current work has identified many sex differences, with some particularly worth noticing. For motor symptoms, a higher proportion of patients reporting dyskinesia has been observed in the female group, which is in line with previous reports [16–18]. The mechanisms underlying this phenomenon have not been clearly elucidated, but pharmacokinetics that can be influenced by body weight has been noted to be partially responsible [19]. The female group in our study took approximately the same medication dosage but significantly lower body weight than the male group. Therefore, the higher percentage of dyskinesia in females may be attributed to the relatively high medication dosage concerning body weight. This finding suggests a more precise medication adjustment according to the patient’s weight. In clinical practice, such knowledge should be taken into account to avoid dyskinesia and other levodopa-associated complications, especially when treating female patients.

For non-motor symptoms, it is worth noticing that depression and anxiety are two common symptoms of PD patients in our study, with more than 50% of patients submitting positive answers for these two questions. When compared between sexes, not only did the female group report significantly more depression and anxiety, they also presented a higher severity of depression which is indicated by the BDI score. Further analysis and research are needed to ascertain this correlation. Nevertheless, undoubtedly there is an overall high prevalence rate of self-identified depression and anxiety in the PD population, and women are more afflicted by these two symptoms than males. Efforts to address depression and anxiety should now be undertaken, especially in female patients.

A remote PRO measurement has been applied in this study through a self-developed smartphone-based platform. This self-management platform provides a new model of clinical management, where patients can report their symptoms remotely and easily with just a click on their smartphones. The timely PROs can ensure the continuity of clinical care outside the clinic and serve as complementary information to support clinicians in decision making. It can also be a potential surrogate when in-person evaluation is not feasible, such as in the COVID-19 pandemic. Most importantly, patients have shown high interest in telemedicine, as a recent survey has identified [20]. Moreover, this platform also opens up a new as well as a feasible opportunity for clinical observation and longitudinal tracking of patient’s behavior and clinical characteristics, including this preliminary work on sex differences, which can lead us to discover pathogenesis, identify critical information, evaluate medication, etc.

The current study still has some limitations. It might be criticized that our data is collected remotely, and the results may not be as solid as those building on data collected by specialist supervision and evaluation at clinics. Actually, previous studies have already reported a high agreement rate between remote and in-person assessments [21, 22]. We cannot exclude inconsistence between the two approaches. For instance, for the question ‘Have you presented a slowness in movement (bradykinesia)?’, there is 9.1% of patients answering ‘NO’. Even though they have been priorly clinically diagnosed as PD by movement disorder specialists at the clinic, they may still deny feeling bradykinesia due to an insensitivity to this particular symptom. Nevertheless, the data is a true reflection of patients’ honest opinions, which is of great clinical significance by itself. If the extrapolation building on PRO is in line with the results in previous works which were conducted under strict supervision at clinics, the data will be further validated. Moreover, many important clinical evaluations, such as Hoehn and Yahr staging, require at-site clinical specialists’ evaluation. Due to the use of smartphone to collect information remotely in this study, unfortunately we cannot collect certain evaluation data, which is a limitation of remote study design. In addition, as this is a pilot cross-sectional study, we did not have enough data to investigate the differences in progression, which needs to and will be answered by further tracking PROs through our smartphone-based platform.

## Conclusions

In conclusion, our results revealed sex differences in PROs of PD and demonstrated the feasibility of remote PRO measurement in PD patients through a smartphone-based platform, which has great significance in guiding further mHealth research and improving personalized clinical care.

### Electronic supplementary material

Below is the link to the electronic supplementary material.

Supplementary Material 1

## Data Availability

The datasets used and/or analyzed during the current study are available from the corresponding author on reasonable request.

## Abbreviations

BDI: Beck Depression Inventory
BMI: Body mass index
LED: Levodopa equivalent dose
MDS-UPDRS: Movement Disorder Society-Sponsored Revision of the Unified Parkinson’s Disease Rating Scale
NMSS: Non-motor Symptoms Scale
PD: Parkinson’s disease
PDQ-8: Parkinson Disease Questionnaire 8
PRO: Patient-reported outcome
RBDSQ: REM Sleep Behavior Disorder Screening Questionnaire
SD: Standard deviation
