# Supplementary material for: Using a smartphone-based self-management platform to study sex differences in Parkinson’s disease: multicenter, cross-sectional pilot study
Source: BMC Med Inform Decis Mak. 2024 Jun 21;24:176. doi: 10.1186/s12911-024-02569-1 (PMC11191196; doi:10.1186/s12911-024-02569-1)
Supplement: Supplementary file 1 — Supplementary Material 1 [file 12911_2024_2569_MOESM1_ESM.docx]

**Supplementary Table 1. Basic Clinical Characteristics, Clinical Questionnaire Scores and LED by Depression**

| Variables | patients with depression | patients without depression | p value |
| --- | --- | --- | --- |
| Age, year, mean ± std | 60.34±11.45 | 60.41±9.93 | 0.857 |
| Sex, % | 47.37% | 64.71% | 0.028 |
| MDS-UPDRS IB, score, mean ± std | 8.05±4.81 | 4.25±3.22 | <0.001 |
| MDS-UPDRS II, score, mean ± std | 14.48±6.83 | 8.32±4.87 | <0.001 |
| NMSS, score, mean ± std | 26.17±21.81 | 4.60±5.18 | 0.061 |
| BDI, score, mean ± std | 18.47±8.16 | 5.13±3.01 | <0.001 |
| RBDSQ, score, mean ± std | 4.85±3.73 | 3.33±2.46 | 0.029 |
| PDQ-8, score, mean ± std | 11.32±6.34 | 4.22±3.58 | <0.001 |
| LED, mean ± std | 515.80±503.18 | 298.42±240.36 | 0.004 |

Abbreviations: PDQ-8: Parkinson Disease Questionnaire 8; BDI: Beck Depression Inventory; NMSS: Non-motor Symptoms Scale; LED: levodopa equivalent dose; MDS-UPDRS: Movement Disorder Society-Sponsored Revision of the Unified Parkinson’s Disease Rating Scale; RBDSQ: REM Sleep Behavior Disorder Screening Questionnaire.

Note: patients with depression: BDI score≥10; patients without depression: BDI<10. Two-tailed p values are presented, and differences were considered statistically significant at p<0.05.

**Supplementary Table 2. PROs by Depression**

| parameter | patients with depression (%) | patients without depression (%) | p value |
| --- | --- | --- | --- |
| Bradykinesia | 97.75 | 89.55 | 0.030 |
| Rigidity | 86.81 | 75.00 | 0.060 |
| Tremor | 78.89 | 78.79 | 0.988 |
| Freezing of Gait | 57.47 | 36.92 | 0.012 |
| Wearing-off | 54.76 | 25.86 | 0.001 |
| Dyskinesia | 34.44 | 22.41 | 0.118 |
| Frequent Falls | 12.79 | 14.93 | 0.703 |
| Posture Instability | 74.44 | 48.48 | 0.001 |
| Better Mobility After Sleep | 63.64 | 63.79 | 0.985 |
| Disease Progression | 77.65 | 55.17 | 0.004 |
| Need Wheelchair | 5.38 | 4.41 | 0.781 |
| Headache | 25.00 | 4.92 | 0.001 |
| Blurred Vision | 55.06 | 27.69 | 0.001 |
| Hyposmia | 47.13 | 43.10 | 0.634 |
| Lisp | 34.78 | 16.92 | 0.013 |
| Drooling | 43.68 | 23.53 | 0.009 |
| Stridor | 10.00 | 4.69 | 0.233 |
| Dyspnea | 7.69 | 4.55 | 0.425 |
| Palpitation | 48.81 | 20.63 | <0.001 |
| Body Pain | 54.65 | 30.30 | 0.003 |
| Humpback | 22.34 | 20.59 | 0.789 |
| Trunk Leaning Forward | 53.19 | 36.76 | 0.039 |
| Scoliosis | 30.85 | 8.82 | 0.001 |
| Hidrosis | 57.78 | 32.31 | 0.002 |
| Anorexia | 31.87 | 10.45 | 0.002 |
| Nausea | 4.35 | 7.58 | 0.388 |
| Difficulty in Swallowing | 21.11 | 15.38 | 0.367 |
| Diarrhea | 3.33 | 5.97 | 0.429 |
| Constipation | 54.74 | 48.44 | 0.435 |
| Fecal Incontinence | 5.26 | 4.55 | 0.837 |
| Dysuria | 14.44 | 8.96 | 0.297 |
| Frequent Urination | 37.21 | 26.15 | 0.151 |
| Urinal Incontinence | 46.81 | 35.82 | 0.164 |
| Hypersexuality | 10.96 | 3.45 | 0.108 |
| Impotence | 11.59 | 6.90 | 0.367 |
| Edema of Lower Extremities | 24.18 | 12.12 | 0.058 |
| Livedo Reticularis of Lower Extremities | 17.86 | 4.62 | 0.014 |
| Hypotension | 26.98 | 18.64 | 0.274 |
| Postural Dizziness | 43.33 | 22.22 | 0.007 |
| Weight Loss | 43.48 | 25.76 | 0.022 |
| Weak | 85.11 | 58.46 | <0.001 |
| Fatigue | 83.52 | 56.25 | <0.001 |
| Daytime Sleepiness | 41.57 | 22.73 | 0.014 |
| Insomnia | 41.86 | 18.75 | 0.003 |
| Restless Leg Syndrome | 32.94 | 8.20 | <0.001 |
| Sleep Disorders in the Past | 45.05 | 36.07 | 0.270 |
| Sleep Disorders Still Persists | 32.53 | 22.58 | 0.188 |
| Depression | 64.37 | 28.33 | <0.001 |
| Anxiety | 79.78 | 43.33 | <0.001 |
| Apathy | 50.00 | 21.21 | <0.001 |
| Compulsion | 4.49 | 1.54 | 0.307 |
| Hallucination | 23.33 | 6.06 | 0.004 |
| Memory Deterioration | 77.17 | 69.35 | 0.278 |
| Mental Problems | 45.56 | 16.67 | <0.001 |

Patients were asked to indicate whether they are afflicted or not by the particular symptom by submitting ‘Yes’ or ‘No’ as their answer, and the percentage of positive answers by depression is shown here. patients with depression: BDI score≥10; patients without depression: BDI<10.

**Supplementary Table 3. Basic Clinical Characteristics, Clinical Questionnaire Scores and LED by NMSS score**

| Variables | patients with  high NMSS score | patients with  low NMSS score | p value |
| --- | --- | --- | --- |
| Age, year, mean ± std | 62.67±12.37 | 59.43±12.20 | 0.053 |
| Sex, male, % | 51.43% | 58.16% | 0.354 |
| MDS-UPDRS IB, score, mean ± std | 9.86±5.70 | 4.95±3.16 | 0.001 |
| MDS-UPDRS II, score, mean ± std | 15.38±9.47 | 9.50±5.14 | 0.016 |
| NMSS, score, mean ± std | 62.14±35.78 | 11.61±8.14 | <0.001 |
| BDI, score, mean ± std | 19.50±4.95 | 8.22±5.65 | 0.043 |
| RBDSQ, score, mean ± std | 4.83±4.79 | 4.44±2.78 | 0.822 |
| PDQ-8, score, mean ± std | 14.33±6.37 | 7.04±4.85 | <0.001 |
| LED, mean ± std | 585.93±361.21 | 477.81±336.18 | 0.032 |

Abbreviations: PDQ-8: Parkinson Disease Questionnaire 8; BDI: Beck Depression Inventory; NMSS: Non-motor Symptoms Scale; LED: levodopa equivalent dose; MDS-UPDRS: Movement Disorder Society-Sponsored Revision of the Unified Parkinson’s Disease Rating Scale; RBDSQ: REM Sleep Behavior Disorder Screening Questionnaire.

Note: patients with high NMSS score: NMSS score above average; patients with low NMSS score: NMSS score below average. Two-tailed p values are presented, and differences were considered statistically significant at p<0.05.

**Supplementary Table 4. PROs by NMSS score**

| parameter | patients with high NMSS score (%) | patients without low NMSS score (%) | p value |
| --- | --- | --- | --- |
| Bradykinesia | 98.57 | 86.86 | 0.006 |
| Rigidity | 89.71 | 80.33 | 0.094 |
| Tremor | 67.19 | 59.40 | 0.292 |
| Freezing of Gait | 70.31 | 35.56 | <0.001 |
| Wearing-off | 70.97 | 35.07 | <0.001 |
| Dyskinesia | 45.31 | 23.31 | 0.002 |
| Frequent Falls | 16.67 | 13.08 | 0.497 |
| Posture Instability | 81.54 | 57.14 | 0.001 |
| Better Mobility After Sleep | 59.02 | 61.67 | 0.730 |
| Disease Progression | 80.00 | 48.24 | 0.001 |
| Need Wheelchair | 17.39 | 7.00 | 0.055 |
| Headache | 23.91 | 9.47 | 0.021 |
| Blurred Vision | 63.41 | 35.42 | 0.002 |
| Hyposmia | 55.74 | 39.84 | 0.040 |
| Lisp | 60.00 | 17.02 | <0.001 |
| Drooling | 47.73 | 17.89 | <0.001 |
| Stridor | 17.07 | 4.30 | 0.013 |
| Dyspnea | 22.73 | 2.06 | <0.001 |
| Palpitation | 55.26 | 27.55 | 0.002 |
| Body Pain | 59.57 | 29.29 | <0.001 |
| Humpback | 36.17 | 17.82 | 0.014 |
| Trunk Leaning Forward | 55.32 | 33.66 | 0.012 |
| Scoliosis | 23.40 | 18.81 | 0.518 |
| Hidrosis | 51.22 | 31.25 | 0.027 |
| Anorexia | 35.56 | 5.15 | <0.001 |
| Nausea | 15.56 | 4.17 | 0.019 |
| Difficulty in Swallowing | 45.45 | 8.00 | <0.001 |
| Diarrhea | 11.36 | 3.06 | 0.047 |
| Constipation | 74.63 | 43.80 | <0.001 |
| Fecal Incontinence | 8.70 | 2.16 | 0.029 |
| Dysuria | 25.58 | 5.10 | <0.001 |
| Frequent Urination | 47.62 | 23.08 | 0.004 |
| Urinal Incontinence | 58.82 | 29.20 | <0.001 |
| Hypersexuality | 12.82 | 4.76 | 0.110 |
| Impotence | 23.68 | 12.05 | 0.102 |
| Edema of Lower Extremities | 19.57 | 10.42 | 0.134 |
| Livedo Reticularis of Lower Extremities | 16.67 | 5.15 | 0.026 |
| Hypotension | 33.33 | 10.39 | 0.004 |
| Postural Dizziness | 41.94 | 21.21 | 0.003 |
| Weight Loss | 36.96 | 18.56 | 0.017 |
| Weak | 82.61 | 61.62 | 0.011 |
| Fatigue | 78.57 | 63.44 | 0.081 |
| Daytime Sleepiness | 46.67 | 26.60 | 0.019 |
| Insomnia | 51.11 | 24.21 | 0.002 |
| Restless Leg Syndrome | 42.11 | 19.10 | 0.007 |
| Sleep Disorders in the Past | 67.16 | 29.77 | <0.001 |
| Sleep Disorders Still Persists | 60.98 | 19.35 | <0.001 |
| Depression | 72.73 | 32.31 | <0.001 |
| Anxiety | 75.38 | 48.51 | <0.001 |
| Apathy | 59.09 | 19.78 | <0.001 |
| Compulsion | 4.44 | 2.08 | 0.431 |
| Hallucination | 45.45 | 8.21 | <0.001 |
| Memory Deterioration | 83.58 | 64.84 | 0.006 |
| Mental Problems | 59.09 | 14.43 | <0.001 |

Patients were asked to indicate whether they are afflicted or not by the particular symptom by submitting ‘Yes’ or ‘No’ as their answer, and the percentage of positive answers by NMSS score is shown here. patients with high NMSS score: NMSS score above average; patients with low NMSS score: NMSS score below average.

**Supplementary Table 5. Linear regression analysis between LED and clinical questionnaire scores.**

| Variables | male group  β (p value) | female group  β (p value) |
| --- | --- | --- |
| MDS-UPDRS IB | 3.35 (0.557) | 10.17 (0.140) |
| MDS-UPDRS II | **8.23 (0.032)** | 7.02 (0.131) |
| NMSS | 2.27 (0.098) | **3.30 (<0.001)** |
| BDI | 10.47 (0.113) | 8.68 (0.191) |
| RBDSQ | 4.70 (0.635) | **28.27 (0.007)** |
| PDQ-8 | **12.03 (<0.001)** | **16.54 (<0.001)** |

Abbreviations: PDQ-8: Parkinson Disease Questionnaire 8; BDI: Beck Depression Inventory; NMSS: Non-motor Symptoms Scale; LED: levodopa equivalent dose; MDS-UPDRS: Movement Disorder Society-Sponsored Revision of the Unified Parkinson’s Disease Rating Scale; RBDSQ: REM Sleep Behavior Disorder Screening Questionnaire.
